# Papillary intralymphatic angioendothelioma of the spleen in a young adult: case report and literature review

**DOI:** 10.3389/fonc.2026.1801536

**Published:** 2026-04-13

**Authors:** Nan Liu, Jie He, Wangwang Liu, Yifan Wang

**Affiliations:** 1 Department of Translational Medicine and Clinical Research, Sir Run Run Shaw Hospital, Zhejiang University School of Medicine, Hangzhou, China; 2 Department of Radiology, Sir Run Run Shaw Hospital, Zhejiang University School of Medicine, Hangzhou, China; 3 Department of Pathology, Sir Run Run Shaw Hospital, Zhejiang University School of Medicine, Hangzhou, China; 4 Department of General Surgery, Sir Run Run Shaw Hospital, Zhejiang University School of Medicine, Hangzhou, China; 5 National Engineering Research Center of Innovation and Application of Minimally Invasive Instruments, Hangzhou, China; 6 Zhejiang Provincial Key Laboratory of Laparoscopic Technology, Sir Run Run Shaw Hospital, Zhejiang University School of Medicine, Hangzhou, China

**Keywords:** cancer surgery, case report, diagnosis, papillary intralymphatic angioendothelioma, spleen, vascular neoplasm, young adult

## Abstract

**Background:**

Papillary intralymphatic angioendothelioma (PILA) is a rare low-grade malignant vascular tumor, typically occurring in the skin and subcutaneous tissues of children. Primary splenic involvement is extremely uncommon. Its imaging features often resemble those of benign splenic lesions such as hemangioma or hamartoma, leading to frequent misdiagnosis. This case highlights the diagnostic challenge and emphasizes the importance of considering PILA in the differential diagnosis of splenic masses, particularly in young patients, due to its potential for local recurrence.

**Case presentation:**

A 21-year-old male presented with a 2-month history of mild left upper abdominal heaviness. He had no fever, pain, trauma history, or hematologic disorders. Systemic symptoms were absent, and tumor markers were normal. Enhanced abdominal CT and MRI revealed a solitary, large heterogeneous mass occupying most of the spleen, initially suggestive of hemangioma or hamartoma. The patient underwent laparoscopic total splenectomy. Following resection, the normal splenic tissue was fragmented into approximately 2 × 2 cm pieces and autotransplanted into the omentum. Final histopathological examination confirmed primary splenic PILA. The postoperative course was uneventful, and the patient was discharged after one week. He remained disease-free at 6-month follow-up.

**Conclusions:**

This report illustrates that PILA, though rare, should be included in the differential diagnosis of solitary vascular splenic masses in young adults. Accurate diagnosis is crucial as PILA possesses a propensity for local recurrence, guiding appropriate surgical management and postoperative surveillance to prevent disease progression.

## 1 Introduction

Papillary intralymphatic angioendothelioma (PILA), also known as Dabska tumor, was first described in 1969 as a distinct entity affecting the skin and subcutaneous tissues of infants and children (1). It is currently classified by the World Health Organization (WHO) as a rarely metastasizing and borderline vascular neoplasm (2, 3). Histologically, PILA is characterized by papillary endovascular projections lined by atypical endothelial cells that protrude into the lumen, often described as “hobnail” cells, creating a glomerulus-like appearance (4).

While the PILA typically manifests as a slow-growing cutaneous nodule, rare cases have been reported in deeper soft tissues, bone, and unusual anatomical sites such as the tongue and testis (5, 6). Primary vascular tumors of the spleen are uncommon, with hemangiomas being the most frequent benign lesions and angiosarcomas representing the most common primary malignant vascular malignancies (7). The occurrence of PILA in the spleen is an exceedingly rare phenomenon, with scarce cases reported in the published literatures to date (8–11).

The diagnostic challenge lies in distinguishing splenic PILA from other vascular lesions. Radiologically, these tumors lack pathognomonic features and may mimic either benign hemangiomas or highly aggressive angiosarcomas (12). However, whereas angiosarcomas carry a dismal prognosis with rapid metastatic potential, PILA generally exhibits an indolent clinical course and a favorable outcome following complete surgical excision (13).

Herein, we report a case of a young male with a massive primary PILA of the spleen. We detail the clinical presentation, imaging characteristics, and distinct histopathological features, while providing a comprehensive review of the literature regarding the management and prognosis of this rare visceral vascular neoplasm.

## 2 Case presentation

### 2.1 Clinical history

A 21-year-old Asian man sought evaluation at the Department of General Surgery, Sir Run Run Shaw Hospital, Zhejiang University School of Medicine, on June 1, 2025. For approximately two months, he had experienced a mild sensation of heaviness localized to the left upper abdominal quadrant. He reported no accompanying symptoms such as fever, night sweats, unintentional weight loss, or fatigue. His past medical and surgical history was unremarkable, and there was no record of abdominal trauma. He also denied occupational or environmental exposure to vinyl chloride or thorium dioxide—agents known to predispose to hepatic or splenic angiosarcoma. The family history did not reveal malignancy or hematologic/coagulation disorders.

### 2.2 Physical examination

At presentation, the patient’s vital signs were within normal limits (temperature 36.8 °C, blood pressure 138/72 mmHg, heart rate 82 beats/min, respiratory rate 20 breaths/min). Abdominal inspection showed no distention or superficial venous engorgement. On palpation, the spleen was appreciably enlarged, with its lower pole palpable roughly 3 cm below the left costal margin. The splenic margin was smooth and firm without tenderness. No hepatomegaly, ascites, or lymphadenopathy in the cervical, axillary, or inguinal regions was detected.

### 2.3 Laboratory findings

Coagulation tests showed a slight decrease in fibrinogen, while the remaining blood routine, liver and renal function, electrolytes, and tumor markers (e.g., CEA, CA19-9, and AFP) were all within normal limits.

### 2.4 Imaging findings

**Figure 1:**
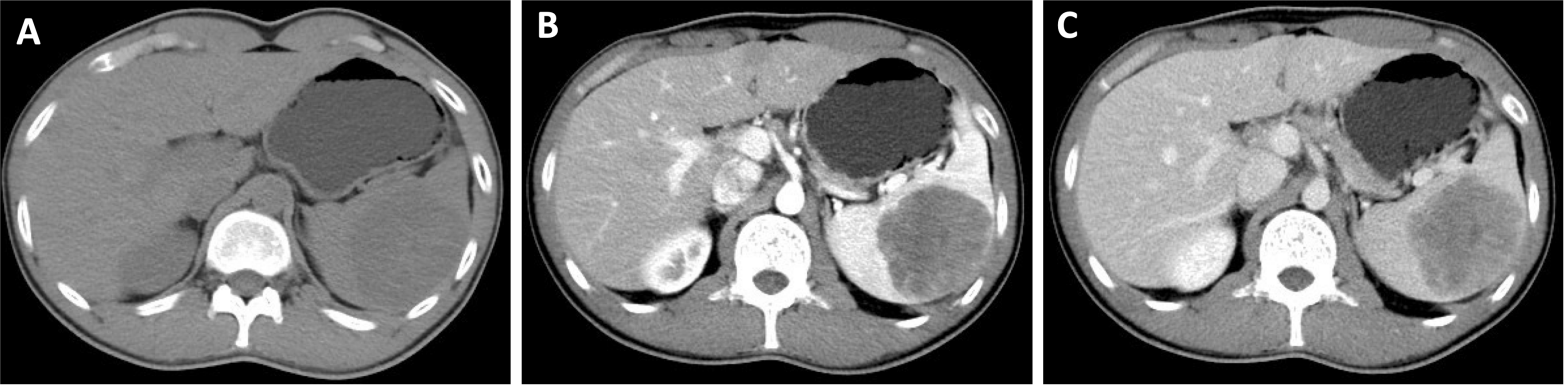
A 21-year-old man with a splenic mass diagnosed as papillary intralymphatic angioendothelioma. A plain abdominal CT scan **(A)** reveals an isodense mass in the spleen, showing mild enhancement in the arterial phase **(B)** and further enhancement in the venous phase **(C)**.

**Figure 2:**
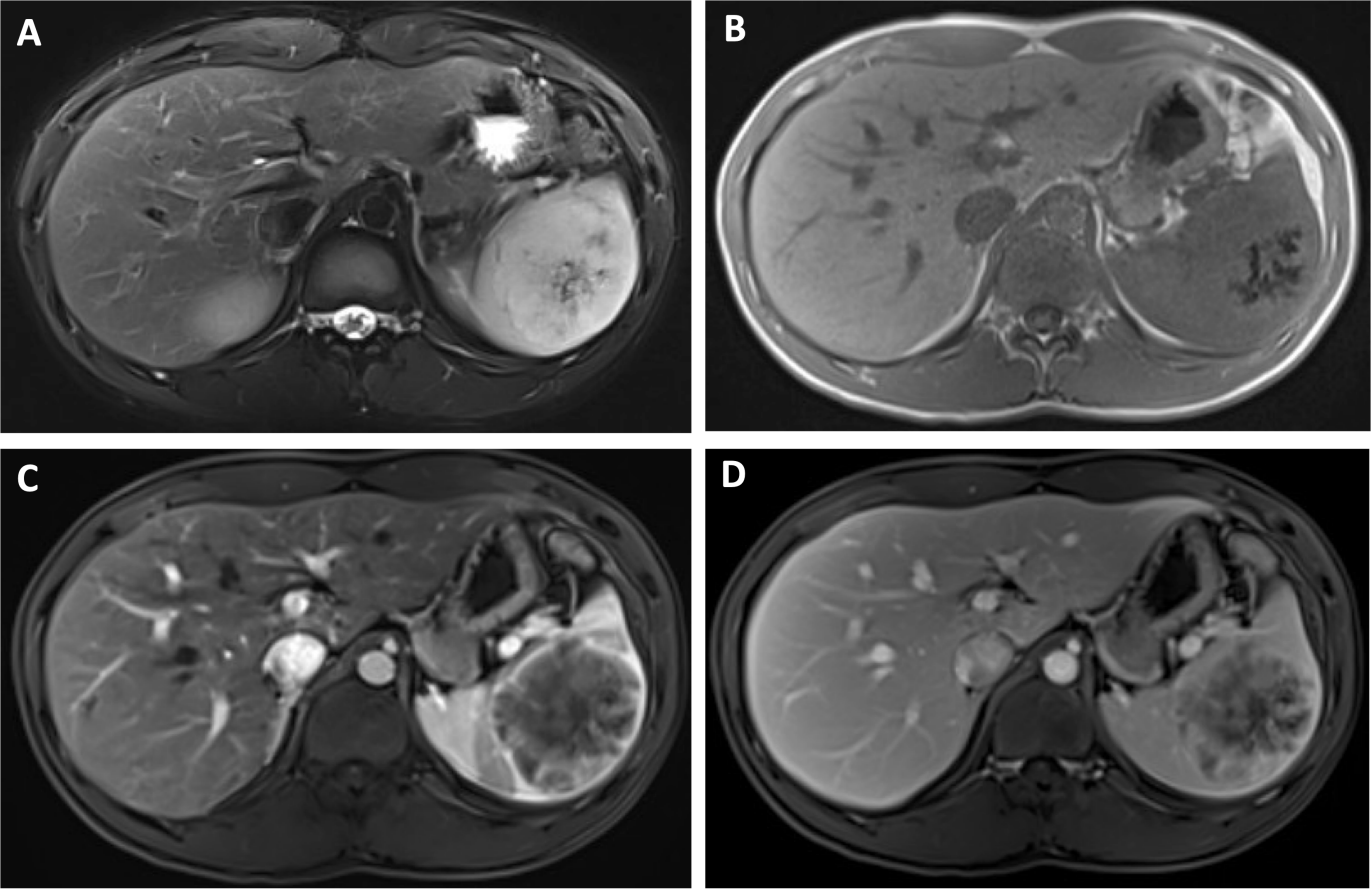
Axial MRI demonstrates that the mass appears slightly hyperintense on T2-weighted imaging **(A)** and slightly hypointense on T1-weighted imaging **(B)**. On contrast-enhanced sequences, the lesion shows marked heterogeneous enhancement during the arterial phase **(C)**, followed by progressive fill-in during the venous phase **(D)**.

Contrast-enhanced CT of the upper abdomen showed a mildly enlarged spleen containing a solitary, well-circumscribed, slightly lobulated hypodense mass in the mid–lower pole, measuring approximately 5.8 × 6.6 cm (**Figure 1**). The lesion showed heterogeneous, mild-to-moderate enhancement after contrast administration without definite central necrosis or calcification. Subsequent contrast-enhanced MRI depicted that the lesion exhibited mixed but predominantly slightly hypointense T1 and slightly hyperintense T2 signal, without convincing diffusion restriction. Dynamic contrast-enhanced sequences showed markedly heterogeneous, progressive enhancement with irregular non-enhancing foci, while the surrounding splenic parenchyma and adjacent organs remained normal (**Figure 2**). Overall, radiologic features were most consistent with a benign splenic mass, with a differential diagnosis of splenic hamartoma versus hemangioma, and no imaging evidence of extra-splenic spread.

### 2.5 Surgical intervention

Given the considerable size of the lesion, the potential risk of spontaneous splenic rupture, and the uncertainty of its nature, surgical management was deemed necessary. The patient underwent a laparoscopic total splenectomy with autologous splenic implantation under general anesthesia on June 3, 2025.

During the procedure, a predominantly cystic mass measuring roughly 8 cm in diameter was noted along the mid-to-upper portion of the spleen, protruding outward. The lesion was encapsulated, smooth, and showed a well-defined interface with adjacent tissues. Following splenic removal, the tumor-containing segment was resected ex vivo, and the residual uninvolved splenic parenchyma was cut into fragments of approximately 2 × 2 cm. These tissue pieces were subsequently implanted into pockets fashioned within the greater omentum to facilitate autologous splenic transplantation.

### 2.6 Pathology and immunohistochemistry

**Figure 3:**
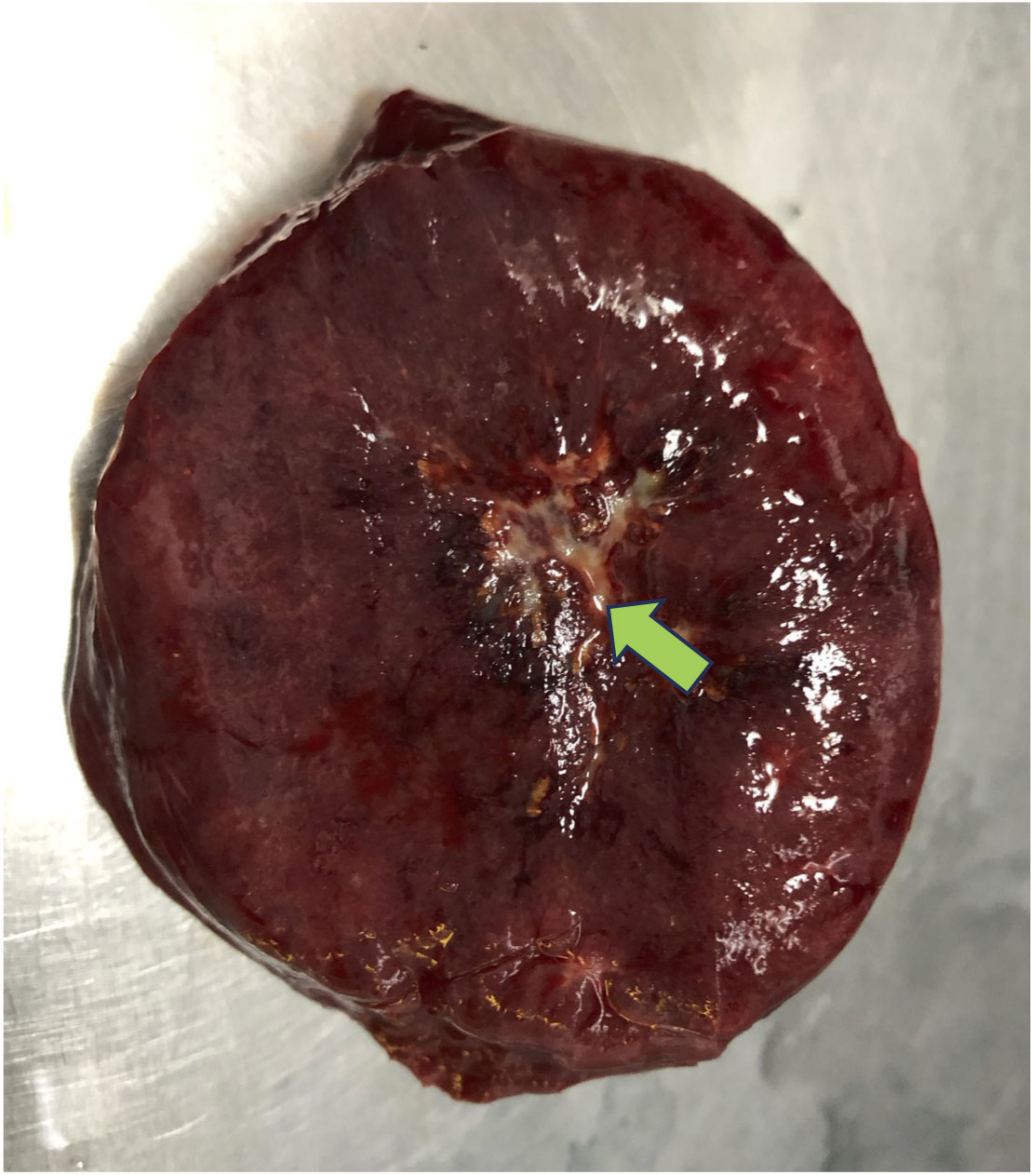
Gross examination of the splenectomy specimen reveals an organ measuring 8.5 × 8.0 × 6.0 cm, within it a well-demarcated lesion measuring 7.5 × 6.2 × 5.2 cm was identified. A well-demarcated intraparenchymal lesion measuring 7.5 × 6.2 × 5.2 cm is identified, with a central gray-white to yellow fibrous scar-like area (green arrow).

**Figure 4:**
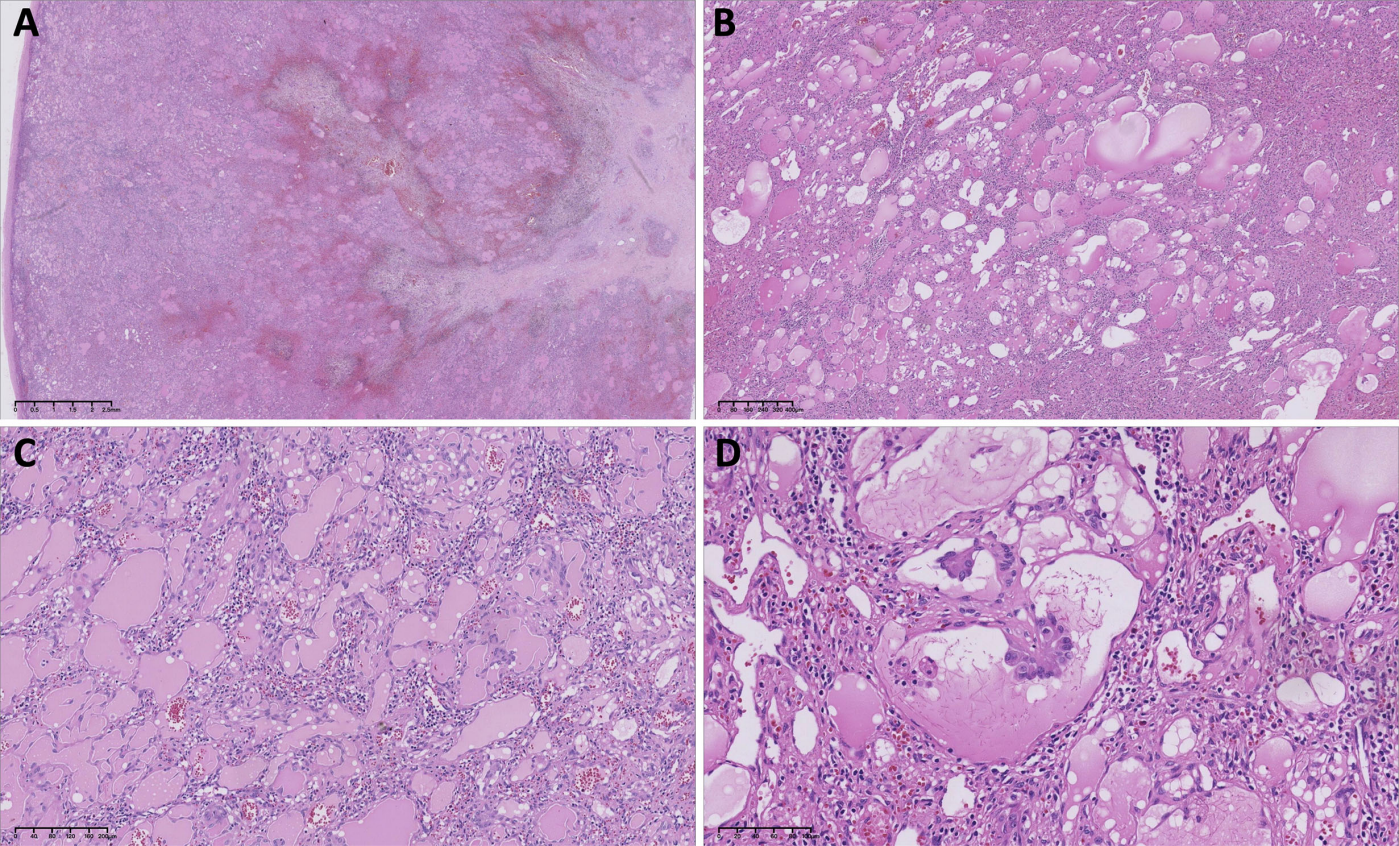
Histopathological examination of the splenic lesion. Low-power view **(A)** shows an ill-defined tumor with a central fibrous scar surrounded by areas of hemorrhage. At 4× magnification **(B)**, the lesion is composed of diffusely proliferating, irregularly shaped vascular channels of variable caliber. At 10× magnification **(C)**, the lumina contain pale eosinophilic secretions resembling lymphatic fluid. At 20× magnification **(D)**, some vascular spaces show intraluminal papillary projections lined by hobnail endothelial cells.

On gross examination, the resected specimen consisted of a portion of spleen containing a circumscribed intraparenchymal mass (**Figure 3**). The spleen measured 8.5 × 8.0 × 6.0 cm, and within it a well-demarcated lesion measuring 7.5 × 6.2 × 5.2 cm was identified. The central portion of the lesion showed a gray white to yellow fibrous scar-like area. Histopathological examination (**Figure 4**) revealed that the tumor was composed of diffusely proliferating vascular channels, some of which contained intraluminal papillary projections lined by hobnail endothelial cells. The stroma exhibited chronic inflammatory cell infiltration with focal hemorrhage and hemosiderin deposition.

Immunohistochemical analysis (**Supplementary Figure 1**) showed that the neoplastic endothelial cells exhibited strong positivity for CD31, CD34, ERG, D2-40, and VEGFR-3, with focal or partial expression of CD8, CD163, and CD68/KP-1. Cytokeratin CK-pan (AE1/AE3), CD21, and HMB45 were negative. Smooth muscle actin highlighted the stromal framework, and the Ki-67 proliferative index was low. Together, these morphologic and immunophenotypic findings supported a diagnosis of PILA (Dabska tumor; ICD-O code 9135/1) arising within the spleen.

### 2.7 Postoperative follow-up

The postoperative course was uneventful, and the patient was discharged on postoperative day 7 (June 9, 2025). Given the low-grade malignant potential of PILA and the achievement of complete surgical excision, no adjuvant radiotherapy or chemotherapy was initiated. The patient has undergone structured surveillance with abdominal ultrasound and CT every three months over a 6-month period, with no evidence of local recurrence or distant metastasis to date. A summary of all published cases of splenic PILA, including the present case, is provided in **Supplementary Table 1**.

## 3 Discussion

Historically, there has been debate regarding the classification of PILA. While initially considered a benign “vegetating hemangioendothelioma”, it is now recognized by the WHO Classification of Tumors of Soft Tissue and Bone as a locally aggressive, rarely metastasizing low-grade malignancy, falling under the umbrella of angiosarcomas or retiform hemangioendotheliomas (14). The tumor has a predilection for the skin and subcutis of the extremities in infants and children. However, adult onset and visceral involvement, although rare, are increasingly recognized. Our patient represents a demographic intersection between the pediatric predilection of the tumor and the adult-onset typically seen in visceral sarcomas.

The etiology of PILA remains obscure. Unlike hepatic angiosarcomas, which are associated with environmental toxins (15), no specific risk factors have been identified for PILA. Recent molecular studies have begun to elucidate the genetic landscape of vascular tumors. Recurrent YAP1 and MAML2 gene rearrangements have been identified in retiform hemangioendothelioma and composite hemangioendothelioma, which exist along a morphologic continuum with PILA. A novel YAP1::MAML2 fusion has recently been described in a case of PILA (16), further supporting a shared genetic basis among these low-grade vascular neoplasms. Emerging evidence has linked PILA to somatic PIK3CA mutations, particularly in the context of PIK3CA-related overgrowth spectrum (PROS). Debelenko et al. reported a case of splenic PILA in a child with PROS harboring a pathogenic PIK3CA variant (c.1357G>A), implicating the PI3K/AKT/mTOR pathway in PILA pathogenesis (17). Building on this, Segal et al. reported targeted treatment with the PI3Kα-selective inhibitor alpelisib for PILA associated with somatic PIK3CA mosaicism, representing a potential therapeutic avenue (18). A recent comprehensive analysis of 118 vascular tumors demonstrated the diagnostic utility of molecular markers such as CAMTA1, FOSB, and TFE3 in differentiating vascular neoplasm subtypes (19). Nevertheless, routine molecular testing is not currently part of standard clinical practice. In our case, genetic sequencing was not performed, but the strong immunoreactivity for VEGFR-3 and D2–40 supports the hypothesis that the tumor arises from precursor cells with a lymphatic phenotype (20).

Diagnosing splenic PILA preoperatively is notoriously difficult. Its radiological features are non-specific and overlap significantly with a variety of vascular lesion (21). (1) Cavernous hemangioma: The most common benign splenic tumor. On MRI, it typically demonstrates the classic “light-bulb” T2 hyperintensity and peripheral puddling pattern of contrast enhancement. In contrast, PILA may lack uniform T2 hyperintensity due to its higher cellular density and papillary architecture (22). (2) Splenic hamartoma: While exhibiting overlapping T2 hyperintensity, it is distinguished by prolonged enhancement and a tendency to become isointense to the splenic parenchyma in the delayed phase, reflecting its red pulp composition. In contrast, PILA typically retains heterogeneous enhancement without equilibrating with the background spleen. Histologically, the definitive discriminator is immunohistochemical CD8 positivity (specific for splenic sinus endothelium) in hamartomas, whereas PILA is consistently CD8-negative and D2–40 positive. (3) Splenic angiosarcoma: The most critical and life-threatening differential diagnosis. Angiosarcomas are highly aggressive, high-grade malignancies with a dismal prognosis (median survival < 6 months) (23). Histologically, they are characterized by solid sheets of anaplastic endothelial cells, high mitotic activity, and extensive necrosis. PILA can be distinguished from angiosarcoma by its well-formed papillary structures, low mitotic activity, and characteristic hobnail endothelial cells. (4) Littoral cell angioma: This benign vascular tumor arises from the littoral cells of the splenic sinusoids. Immunohistochemically, littoral cell angiomas show a CD31^+^/CD68^+^ phenotype, whereas PILA typically expresses CD31^+^, CD34^+^, and D2-40^+^ markers and is usually negative for CD68 (24). (5) Kaposi sarcoma: More frequently encountered in immunocompromised individuals, Kaposi sarcoma demonstrates HHV-8 positivity, a feature consistently absent in PILA, providing an important diagnostic distinction (25). (6) Sclerosing angiomatoid nodular transformation (SANT): This rare benign splenic lesion is composed of angiomatoid nodules within a dense sclerotic stroma and characteristically displays a mixed vascular immunophenotype with three distinct channel types (CD34+/CD8−/CD31+, CD34−/CD8+/CD31+, and CD34−/CD8−/CD31+). This triple immunophenotype readily distinguishes SANT from PILA, which exhibits a lymphatic phenotype (D2-40+, VEGFR-3+) (26).

A key histopathologic hallmark of PILA is its “glomeruloid” or papillary architecture. The endothelial cells exhibit classic hobnail morphology, characterized by scant cytoplasm and nuclei protruding into the vascular lumen (4, 27). The differentiation between PILA and retiform hemangioendothelioma (RH) may at times be challenging; however, RH is typified by elongated, arborizing vascular channels reminiscent of the rete testis, rather than the compact papillary tufts that define PILA. Many pathologists now regard PILA and RH as points along a spectrum of low-grade angiosarcomas (21). Immunophenotypically, PILA shows consistent positivity for vascular markers (CD31, CD34, Factor VIII) as well as lymphatic markers (D2-40, VEGFR-3) (28). This lymphatic profile serves as a key discriminator from capillary hemangiomas.

Given the exceptional rarity of splenic PILA, formal treatment guidelines have not been defined. Evidence extrapolated from cutaneous PILA suggests that complete surgical excision with clear margins remains the preferred therapeutic approach (2). For splenic involvement, total splenectomy effectively fulfills both diagnostic and therapeutic purposes. In contrast to cutaneous lesions—where wide local excision is feasible—the spleen’s encapsulated structure generally permits complete tumor removal through splenectomy. Careful operative technique is critical, as intraoperative rupture of the mass may predispose the patient to peritoneal dissemination or sarcomatosis. The role of adjuvant therapy continues to be debated. While systemic chemotherapy (e.g., paclitaxel or doxorubicin) is commonly employed in high-grade angiosarcomas (29), such interventions are usually unnecessary for low-grade PILA unless there is documented metastasis, residual disease, or recurrent tumor following surgery.

The prognosis of PILA is generally favorable compared with conventional angiosarcoma. Lymph-node metastases have been reported in a minority of cases, whereas distant metastasis is rare but possible (30). In a review of cutaneous PILA, the local recurrence rate was approximately 10–15%, largely depending on the adequacy of surgical margins (4, 31). Despite the absence of apparent risk factors for recurrence in our patient, a diagnosis of a “malignant” or “borderline” vascular tumor carries a substantial psychological burden for a young adult. In addition, splenectomy results in permanent asplenia and lifelong immunological vulnerability, particularly the risk of overwhelming post-splenectomy infection (32, 33). Hence, ongoing surveillance remains essential. Postoperative management should include regular monitoring of white blood cell counts, timely administration of appropriate vaccines, and careful patient education regarding the need for prompt medical evaluation of any febrile episode. At 6-month follow-up, our patient remains disease-free and without complications; nevertheless, extended follow-up is required both to assess the long-term consequences of splenectomy and to confirm the indolent clinical course of this neoplasm.

## 4 Conclusion

We reported a rare case of primary splenic PILA in a young male. While vascular masses in spleen are frequently benign hemangiomas, clinicians must maintain a high index of suspicion for malignant or borderline entities when masses are large, symptomatic, or radiologically atypical. The diagnosis relies heavily on histopathological identification of papillary “hobnail” endothelial cells and a lymphatic immunophenotype (D2-40+). Total splenectomy appears to be curative in localized disease, with a favorable prognosis compared to other splenic sarcomas. Continued reporting of such cases is essential to establish long-term prognostic data and refine management algorithms for this rare entity.

## Data Availability

The raw data supporting the conclusions of this article will be made available by the authors, without undue reservation.
