# Supplementary figures and images for: Papillary intralymphatic angioendothelioma of the spleen in a young adult: case report and literature review
Source: Front Oncol. 2026 Apr 13;16:1801536. doi: 10.3389/fonc.2026.1801536 (PMC13111061; doi:10.3389/fonc.2026.1801536)

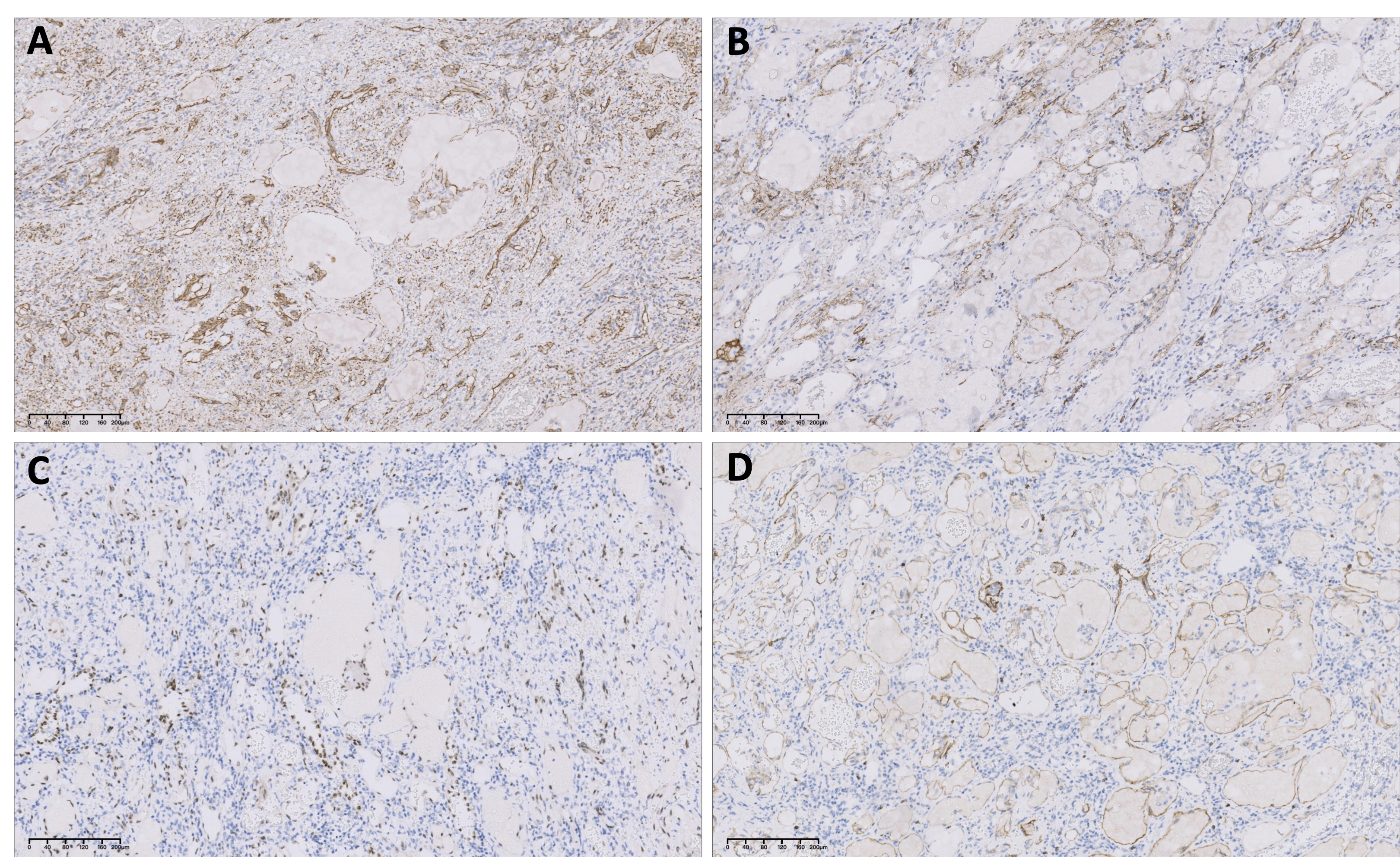

Supplement: Supplementary file 1 [file Image1.tiff]
