# Supplementary material for: Papillary intralymphatic angioendothelioma of the spleen in a young adult: case report and literature review
Source: Front Oncol. 2026 Apr 13;16:1801536. doi: 10.3389/fonc.2026.1801536 (PMC13111061; doi:10.3389/fonc.2026.1801536)
Supplement: Supplementary file 2 [file Table1.docx]

**Table S1. Summary of published cases of splenic papillary intralymphatic angioendothelioma (PILA)**

| Characteristic | Rodgers et al. (2007) | Wang et al. (2023) | Li et al. (2024) | Jiao et al. (2025) | Present case |
| --- | --- | --- | --- | --- | --- |
| Reference number | [10] | [9] | [11] | [8] | Present study |
| Age / Sex | 6 y / F | 47 y / M | 35 y / M | 29 y / F | 21 y / M |
| Geographic origin | USA | China | China | China | China |
| Clinical presentation | left upper quadrant abdominal mass with weight loss and early satiety | Abdominal discomfort | Left upper abdominal discomfort | Left upper abdominal discomfort for 1 month | Left upper abdominal heaviness for 2 months |
| Tumor size | 8.0 × 5.3 cm | Multiple lesions (largest 2.8 cm) | Multiple lesions (size NR) | 6.0 × 5.0 cm | 7.5 × 6.2 × 5.2 cm |
| Imaging findings | It was heterogeneous and  irregularly enhancing on CT | Multiple masses with internal stellate scars, marked hypointensity on T2WI and contrast-enhanced MR. Most lesions showed inhomogeneous enhancement. | Multiple low density lesions without obvious reinforcement were found on CT | Heterogeneous mass with progressive enhancement on CT; increased FDG uptake on PET/CT | Well-circumscribed heterogeneous mass; mixed T1/T2 signal on MRI; progressive enhancement |
| Histological features | Papillary endovascular projections with hobnail endothelial cells | Intraluminal papillary projections lined by hobnail endothelial cells | Papillary intralymphatic proliferation with hobnail endothelial cells | Papillary projections with hobnail endothelial cells within dilated vascular channels | Intraluminal papillary projections lined by hobnail endothelial cells; stromal inflammatory infiltration with hemosiderin deposition |
| IHC profile | CD31+, CD34+, Factor VIII+ | CD31+, CD34+, D2-40+, ERG+ | CD31+, CD34+, ERG+ | CD31+, CD34+, D2-40+, ERG+ | CD31+, CD34+, ERG+, D2-40+, VEGFR-3+; CD8 focal+; CK-pan−, CD21−, HMB45− |
| Molecular profile | Not performed | Not performed | Not performed | Not performed | Not performed |
| Treatment | Total splenectomy | Total splenectomy | Total splenectomy | Total splenectomy | Laparoscopic total splenectomy with autologous splenic implantation |
| Follow-up / Outcome | NED at 12 months | NED at short-term follow-up | NED at 6 months | NED at short-term follow-up | NED at 6 months |

*NR, not reported; NED, no evidence of disease; IHC, immunohistochemistry; US, ultrasonography; CT, computed tomography; MRI, magnetic resonance imaging; PET, positron emission tomography; FDG, fluorodeoxyglucose.*
